# Tumor Necrosis Factor-Alpha Exacerbates Viral Entry in SARS-CoV2-Infected iPSC-Derived Cardiomyocytes

**DOI:** 10.3390/ijms22189869

**Published:** 2021-09-13

**Authors:** Chiu-Yang Lee, Chih-Heng Huang, Elham Rastegari, Vimalan Rengganaten, Ping-Cheng Liu, Ping-Hsing Tsai, Yuan-Fan Chin, Jing-Rong Wu, Shih-Hwa Chiou, Yuan-Chi Teng, Chih-Wei Lee, Yanwen Liang, An-Yu Chen, Shu-Chen Hsu, Yi-Jen Hung, Jun-Ren Sun, Chian-Shiu Chien, Yueh Chien

**Affiliations:** 1 Division of Cardiovascular Surgery, Department of Surgery, Taipei Veterans General Hospital, Taipei 11217, Taiwan; david.lee0501@gmail.com; 2 Institute of Clinical Medicine, School of Medicine, National Yang-Ming University, Taipei 11217, Taiwan; 3 Institute of Preventive Medicine, National Defense Medical Center, Taipei 11217, Taiwan; chin0096@gmail.com (C.-H.H.); d790706d@gmail.com (P.-C.L.); googling0204@gmail.com (Y.-F.C.); pcsppo3zx567@gmail.com (A.-Y.C.); schsu0925@gmail.com (S.-C.H.); 4 Graduate Institute of Medical Sciences, National Defense Medical Center, Taipei 11217, Taiwan; 5 Department of Microbiology and Immunology, National Defense Medical Center, Taipei 11217, Taiwan; 6 Institute of Pharmacology, National Yang-Ming University, Taipei 11217, Taiwan; elham.rastegar@gmail.com (E.R.); vimalanrengganaten@gmail.com (V.R.); figatsai@gmail.com (P.-H.T.); henrywu0228@gmail.com (J.-R.W.); shchiou@vghtpe.gov.tw (S.-H.C.); andrea_chi@hotmail.com (Y.-C.T.); q147555251@gmail.com (C.-W.L.); 7 Department of Medical Research, Taipei Veterans General Hospital, Taipei 11217, Taiwan; yanwen95ktb@gmail.com; 8 School of Medicine, National Yang-Ming Chiao Tung University, Taipei 11217, Taiwan; 9 Centre for Stem Cell Research, Universiti Tunku Abdul Rahman, Kajang 43000, Malaysia; 10 Postgraduate Programme, Department of Preclinical Sciences, Faculty of Medicine and Health Sciences, Universiti Tunku Abdul Rahman, Kajang 43000, Malaysia; 11 Department of Ophthalmology, Taipei Veterans General Hospital, Taipei 11217, Taiwan; 12 Department of Life Sciences and Institute of Genomic Sciences, National Yang-Ming University, Taipei 11217, Taiwan

**Keywords:** SARS-CoV2, cardiomyocytes, TNF-α, induce pluripotent stem cells, SARS-CoV2 pseudovirus, inflammation

## Abstract

The coronavirus disease 2019 (COVID-19) pandemic with high infectivity and mortality has caused severe social and economic impacts worldwide. Growing reports of COVID-19 patients with multi-organ damage indicated that severe acute respiratory syndrome coronavirus 2 (SARS-CoV2) may also disturb the cardiovascular system. Herein, we used human induced pluripotent stem cell (iPSC)-derived cardiomyocytes (iCMs) as the in vitro platform to examine the consequence of SARS-CoV2 infection on iCMs. Differentiated iCMs expressed the primary SARS-CoV2 receptor angiotensin-converting enzyme-II (ACE2) and the transmembrane protease serine type 2 (TMPRSS2) receptor suggesting the susceptibility of iCMs to SARS-CoV2. Following the infection of iCMs with SARS-CoV2, the viral nucleocapsid (N) protein was detected in the host cells, demonstrating the successful infection. Bioinformatics analysis revealed that the SARS-CoV2 infection upregulates several inflammation-related genes, including the proinflammatory cytokine tumor necrosis factor-α (TNF-α). The pretreatment of iCMs with TNF-α for 24 h, significantly increased the expression of ACE2 and TMPRSS2, SASR-CoV2 entry receptors. The TNF-α pretreatment enhanced the entry of GFP-expressing SARS-CoV2 pseudovirus into iCMs, and the neutralization of TNF-α ameliorated the TNF-α-enhanced viral entry. Collectively, SARS-CoV2 elevated TNF-α expression, which in turn enhanced the SARS-CoV2 viral entry. Our findings suggest that, TNF-α may participate in the cytokine storm and aggravate the myocardial damage in COVID-19 patients.

## 1. Introduction

The coronavirus disease 2019 (COVID-19) caused a global pandemic that has progressed rapidly and continues to date, manifesting severe effects through contributing additive factors in specific individuals. Over 93 million people have tested positive for severe acute respiratory syndrome coronavirus-2 (SARS-CoV-2) and close to 2 million deaths worldwide [1]. The preliminary findings of COVID-19 infection have shown pulmonary distress, with symptoms ranging from mild upper respiratory infection to severe pneumonia and acute respiratory distress syndrome [2]. Similar to SARS-CoV, angiotensin-converting enzyme 2 (ACE2) is the primary entry receptor for SARS-CoV2 virus [3]. The inhibition of both ACE2 and the transmembrane protease serine type 2 (TRMPRSS2) receptor (TRMPSS2) was shown to reduce the viral entry into the cells, hence decreasing infection risk and severity [4]. The abundance of ACE2 and TRMPSS2 receptors in the lungs could explain the severity of COVID-19 infection in the pulmonary system. However, ACE-2 and TRMPSS2 receptors are also found in extrapulmonary organs, including the heart and liver [5,6,7]. Accordingly, growing evidence indicates a possible multi-organ damage in patients with COVID-19, including the heart. The expression of ACE-2 and TRMPSS2 receptors on the heart tissue implies a link between SARS-CoV-2 virus and the heart [7]. The detection of the SARS-CoV-2 viral RNA in human autopsy heart tissue further suggests the impact of the infection on the cardiovascular system [8].

Induced pluripotent stem cells (iPSCs) carry the features of embryonic stem cells, including pluripotency and stemness maintenance. The generation of iPSCs can be induced by the forced expression of transcription factors, including Oct4, Sox2, Klf4, and c-Myc, into somatic cells. The development of induced pluripotent stem cell (iPSC) technology has provided opportunities in regenerative medicine, personalized therapy, and patient-specific cell therapy. Due to the differentiation potential and the ability for cell expansion, iPSCs can be differentiated into a variety of cells, including cardiomyocytes [9,10], hepatocytes [11], and dopaminergic neurons [12]. The advances in iPSC technology have also allowed these pluripotent stem cells to differentiate into retinal organoids [13], lung organoids [14], and liver organoids [15]. We have used iPSC-derived retinal organoids that express ACE2 and TMPRSS2 as the platform for investigating the pathogenesis of SARS-CoV2 infection in the retina [16]. Recent reports have shown that human iPSC-derived cardiomyocytes are susceptible to SARS-CoV-2 [17,18,19], and the viral infection can impair the electrical and mechanical functions [18]. However, the consequence of SARS-CoV2 infection on inflammatory cytokines in cardiomyocytes remains unclear.

The development of a strong cytokine storm was reported as the major undesirable consequence in COVID-19 patients [20]. Among the reported cytokines, tumor necrosis factor-alpha (TNF-α) has been associated as a key component of the cytokine storm observed in many COVID-19 patients [21]. The immune cells release various cytokines to respond to the SARS-CoV-2 virus infection, including TNF-α and the members of the interleukin family [22,23]. The accumulations of the cytokines cause high infiltration of immune cells into the heart cells, leading to the heart failure [24]. The TNF-α expression significantly increases during the initial stages of SARS-CoV-2 infection and boosting the expression throughout the infection [21]. Patients with high TNF-α levels commonly require ICU admission [25]. The importance of TNF-α in COVID-19 has been highlighted with a recent clinical trial involving Adalimumab, a TNF-α inhibitor, as a potential treatment for COVID-19 patients (CHICTR2000030089). However, the role of TNF-α in SARS-CoV-2 pathogenesis in the heart needs to be further investigated.

In this study, we examined the impact of the SARS-CoV-2 infection on human iPSC-derived functional cardiomyocytes (iCMs). Interestingly, we found that TNF-α expression is upregulated according to NGS (next-generation sequencing) analysis. To examine whether the upregulation of TNF-α plays a role in viral infection, we assessed the expression of ACE2 and the TMPRSS2, the viral entry receptor proteins involved in the spike protein (S-protein) priming. Under the exposure to TNF-α, the viral entry of SARS-CoV2 pseudovirus was significantly exacerbated in iPSC-derived cardiomyocytes. Collectively, our data indicated that TNF-α increases the susceptibility of iCMs to SARS-CoV2.

## 2. Results

### 2.1. Generation of iPSC-Derived Cardiomyocytes Using Cardiac Induction Protocol

**Figure 1:**
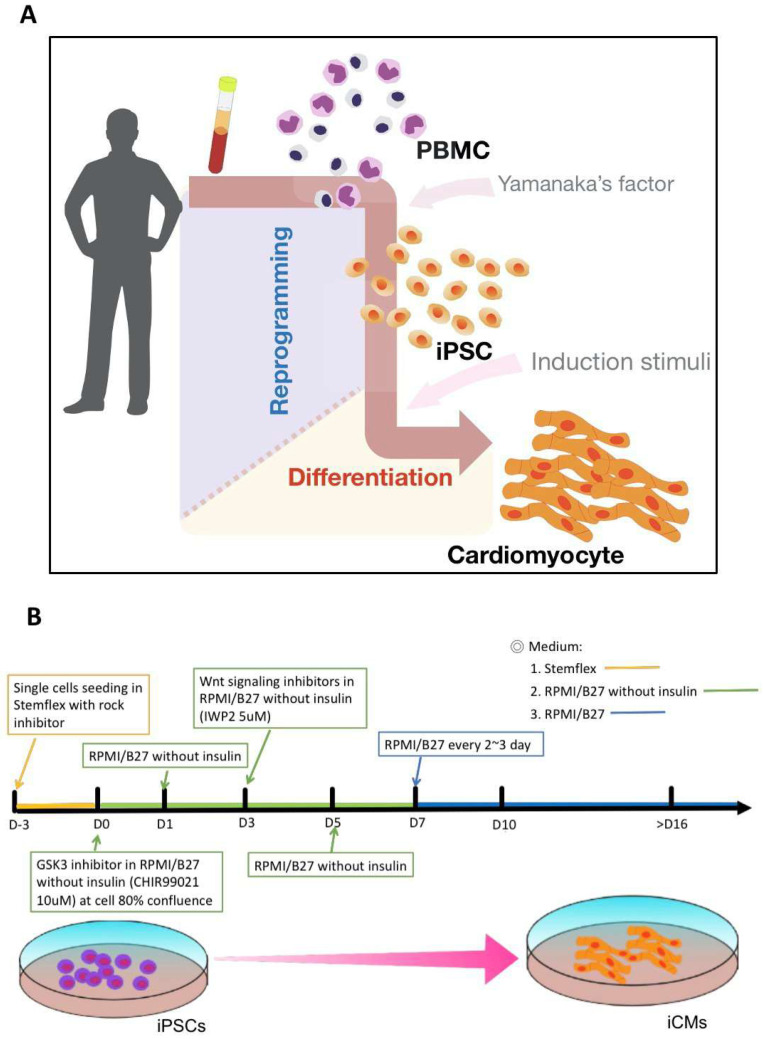
Schemes for hiPSC generation and the differentiation of iCMs. (**A**) Peripheral blood mononuclear cells (PBMCs) were reprogrammed into hiPSCs via transducing the Yamanaka’s factors, Oct4, Sox2, Klf4, and c-Myc. The resulting hiPSCs were then subjected to cardiac induction. (**B**) Functional mature iCMs with beating characteristics can be obtained at 16 days post-induction using the defined protocol of cardiac induction.

In order to initiate this study, we generated human iPSCs from peripheral blood mononuclear cells (PBMCs) via transducing the Yamanaka’s factors, Oct4, Sox2, Klf4, and c-Myc. The resulting human iPSCs were routinely cultivated (Figure 1A), followed by the modulation of Wnt signaling pathway to facilitate the differentiation of hiPSCs into iCMs (Figure 1B) [26], for the stable generation of functional iCMs [9,10].

**Figure 2:**
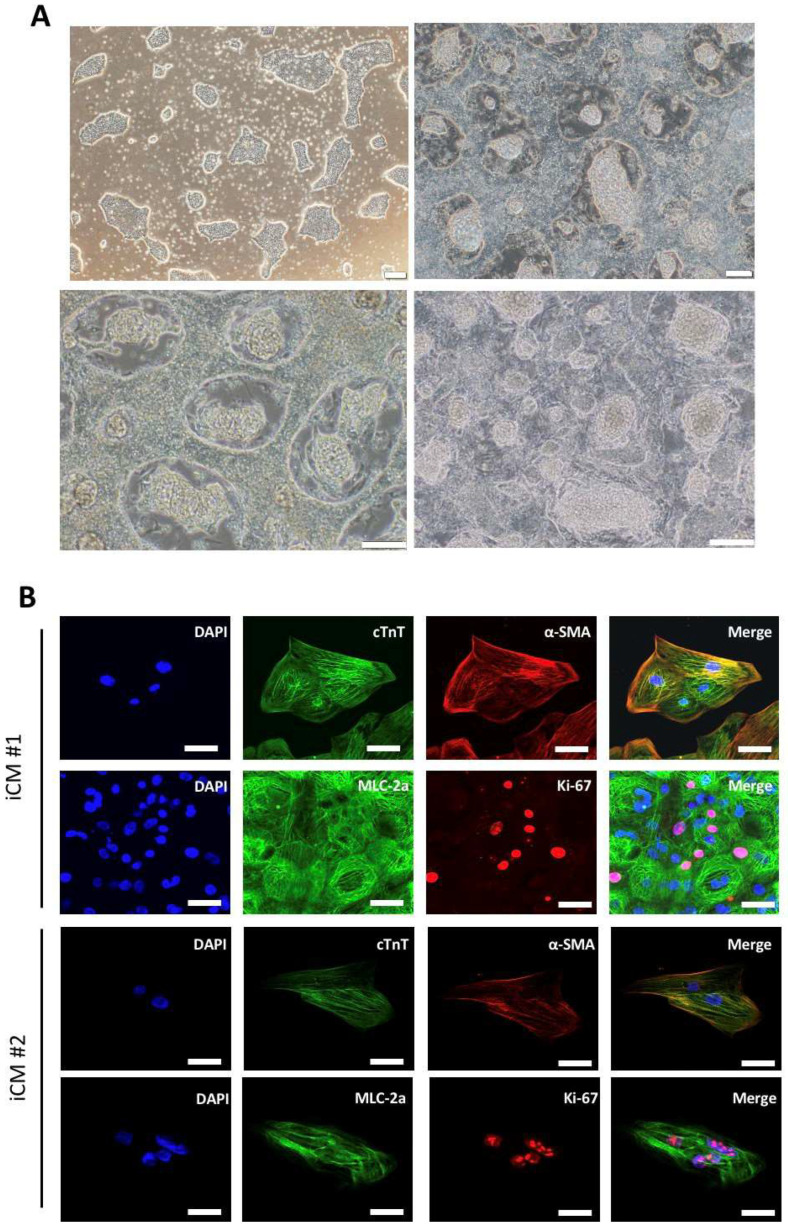
Generation of iCMs after the cardiac induction. (**A**) Distinct cell morphologies during the cardiac induction were shown: hiPSCs (upper left); iCMs (post-induction D12; upper right and lower left); iCMs (post-induction D16; lower right). Scale bar = 200 μm (Upper left) or 100 μm (Upper right, lower left, and lower right). (**B**) Immunofluorescence staining showed the expression of cTnT, α-SMA, MLC-2a, and Ki-67 in iCMs in two generated iCM clones. Scale bar = 20 μm.

Twelve days post-induction, iCMs generated from two independent iPSC clones showed cardiomyocyte morphology and rhythmic contraction similar with iPSCs control group, exhibiting typical embryonic stem cell characteristics (upper left, Figure 2A). Notably, at 16 days post-induction, all iCMs were defined as mature cardiomyocytes and used in the following experiments. To verify the successful differentiation of iPSCs into cardiomyocytes, the immunofluorescence staining demonstrated the presence of mature cardiomyocyte markers in the two generated iCM clones. As shown in Figure 2B, both iCM clones were positively stained for cardiac markers, including cardiac troponin T (cTNT), alpha-smooth muscle actin (α-SMA), myosin regulatory light chain 2, atrial isoform (MLC-2a), and the marker for dividing cells Ki67, validating the typical features of cardiomyocytes.

### 2.2. SARS-CoV2 Viral Infection in iPSC-Derived Cardiomyocytes

**Figure 3:**
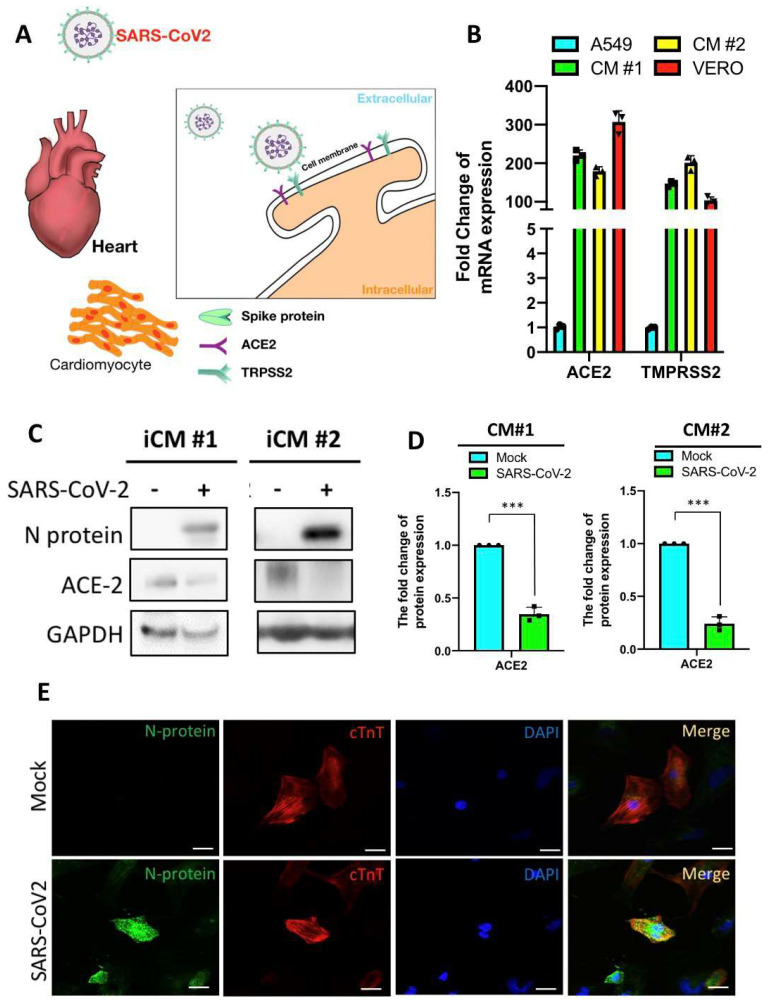
SARS-CoV2 infection in hiPSC-derived cardiomyocytes. (**A**) A proposed scheme for SARS-CoV2 infection in iCMs. (**B**) qRT-PCR showing the expression of ACE2 and TMPRSS2 in iCMs. A549 cells and VERO cells were used as negative- and positive-control cells, respectively. *** *p* < 0.001 vs. A549. (**C**) Western blot of N protein and ACE2 in iCMs with or without SARS-CoV2 infection. (**D**) The quantifications of Western blots. (**E**) Immunofluorescence staining showed the expression of N protein and cTnT in iCMs with or without SARS-CoV2 infection. Scale bar = 20 μm.

As mentioned earlier, SARS-CoV2 possesses high infectivity and pathogenicity. Therefore, we conducted the SARS-CoV2 viral infection in a maximum containment (Biosafety level 4; BSL4) laboratory. It has been well demonstrated that SARS-CoV2 infected mammalian cells via the receptors ACE2 and TMPRSS2 (Figure 3A). We first used quantitative real-time polymerase chain reaction (qRT-PCR) to examine the expression of ACE2 and TMPRSS2 in iCMs. The adenocarcinomic human alveolar basal epithelial cells A549 cells express extremely low expression of ACE2 and TMPRSS2 while the African green monkey kidney cell line VERO cells express high levels of ACE2- and TMPRSS2. Therefore, A549 and VERO cells were used as negative- and positive controls in the following experiments, respectively. Compared with A549 cells and VERO cells, our two generated iCM clones showed abundant expression of both ACE2- and TMPRSS2 (Figure 3B). We next performed Western blot and immunofluorescence staining to assess the consequence of viral infection in iCMs clones. After the exposure of iCMs to SARS-CoV2 for 24 h, the SARS-CoV2 nucleocapsid (N) protein, an abundant RNA-binding protein critical for viral genome packaging, could be robustly detected in the protein extracts of iCMs clones. ACE2 expression was declined after the viral infection, consistent with previous observations by Sharma et al. [18] (Figure 3C,D). We also found the same inclusion of SARS-CoV2 N protein in the host iCMs using immunofluorescence staining (Figure 3E). Collectively, these data suggest iCMs as a feasible platform for SARS-CoV2 infection.

### 2.3. SARS-CoV2 Viral Infection Exacerbated Inflammation-Related Pathways

**Figure 4:**
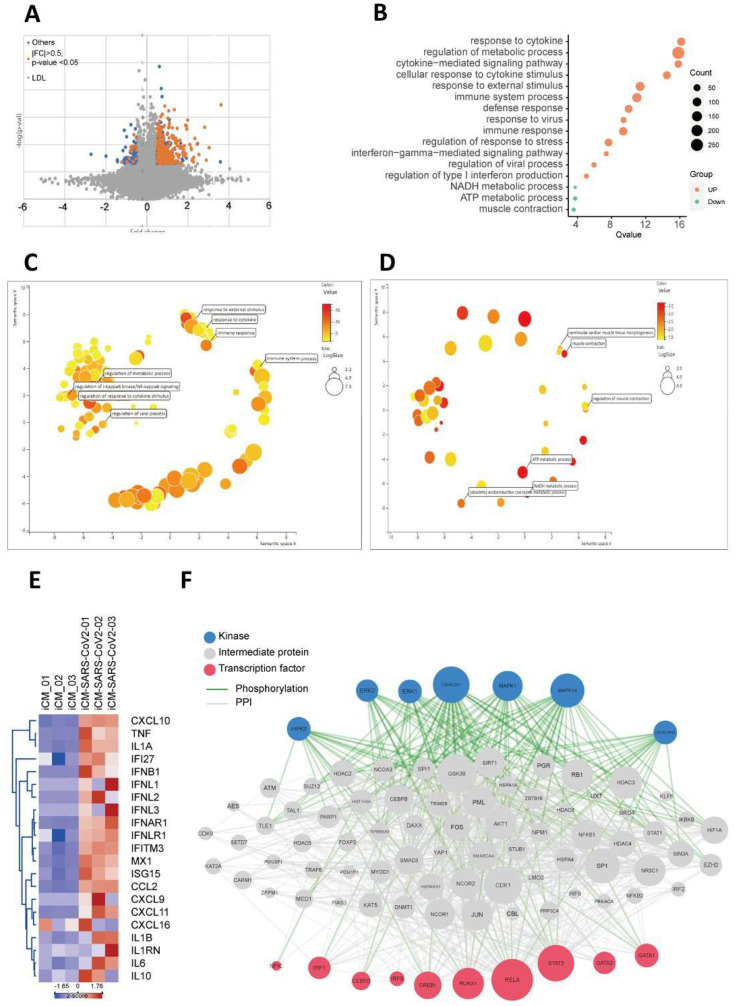
RNA-seq analysis of hiPSC-derived iCMs after SARS-CoV-2 infection. (**A**) Volcano plot showing the genes that were either upregulated or downregulated by SARS-CoV2 infection. (**B**) Enrichment plot showing several pathways that were either enriched or suppressed by SARS-CoV2 infection. Those enriched pathways were related to the regulation of the metabolic pathways, response to cytokine, cytokine-mediated signaling pathway, cellular response to cytokine stimulus, response to external stimulus, immune system process, response to the virus, regulation of response to stress, interferon-gamma-mediated signaling pathway, regulation of the viral process, regulation of type I interferon production. Those pathways downregulated by viral infection included NADH metabolic process, ATP metabolic process, and muscle contraction. (**C**) Validating those pathways that were either downregulated or (**D**) upregulated by SARS-CoV2 using REVIGO semantic similarity-based scatterplots. (**E**) A hierarchical heatmap showed the differentially regulated gene expression patterns of inflammation-related genes in iCMs with or without SARS-CoV2 infection. Red and blue colors represented differentially expressed genes that were upregulated and downregulated, respectively. Many inflammation-related genes were elevated by the viral infection, including the CXCL10, TNF-α, and interleukin 1α. (**F**) eXpression2Kinases network identified the network that consists of various kinases and transcription factors upstream to these identified inflammation-related genes.

To evaluate the effect of SARS-CoV2 infection on the gene expression profiles, RNA was extracted from normal and infected iCMs, followed by genome-wide gene expression array analysis. A volcano plot revealed that a variety of genes were either upregulated or downregulated by the SARS-CoV2 viral infection. A volcano plot demonstrated the modulation of gene expression upon the SARS-CoV2 infection (Figure 4A). Enrichment plot shows the upregulation of genes involved in the regulation of the metabolic pathways, response to cytokine, cytokine-mediated signaling pathway, cellular response to cytokine stimulus, response to external stimulus, response to the virus, immune response, regulation of response to stress, interferon-gamma-mediated signaling pathway, regulation of the viral process, and the regulation of type I interferon production. Furthermore, signaling pathways involved in NADH and ATP metabolism as well as muscle contraction were downregulated. (Figure 4B). Consistent with the Enrichment plot data, REVIGO semantic similarity-based scatterplots further validated the modulation of the aforementioned pathways (Figure 4C,D). Considering the role of cytokines in COVID-19 pathogenesis, we subsequently focused on the upregulated genes associated with inflammatory responses to SARS-CoV2 infection. The expression profiles of these enriched genes were shown in the hierarchical heatmap, including CXCL10, tumor necrosis factor-α (TNF-α), interleukin 1α, IFI27, etc. (Figure 4E). Since genome-wide mRNA profiling only provides the information of the global mRNA levels, Chen et al. developed the network tool Expression2Kinases (X2K) to infer the upstream regulatory networks of differentially expressed genes [27]. X2K combines transcription factor enrichment analysis, protein–protein interaction network expansion with kinase enrichment analysis. Integrated functions of X2K allows the generation of inferred networks, including transcription factors, proteins, and kinases, which are predicted to regulate the profiles of differentially expressed genes. In this study, we focused on the upregulated inflammation-related genes and subjected them to the X2K analysis. The inferred networks of SARS-CoV2-upregulated inflammation-related genes were predicted to be made up of kinases (HIPK2, ERK2, ERK1, CSNK2A1, MAPK1, and MAPK14) and transcription factors (NFIC, IRF1, CEBPD, IRF8, CREB1, RUNX1, RELA, STAT3, GATA2, and GATA1) Figure 4F.

### 2.4. TNF-α Treatment Exacerbated SARS-CoV2 Pseudoviral Infection in iCMs

**Figure 5:**
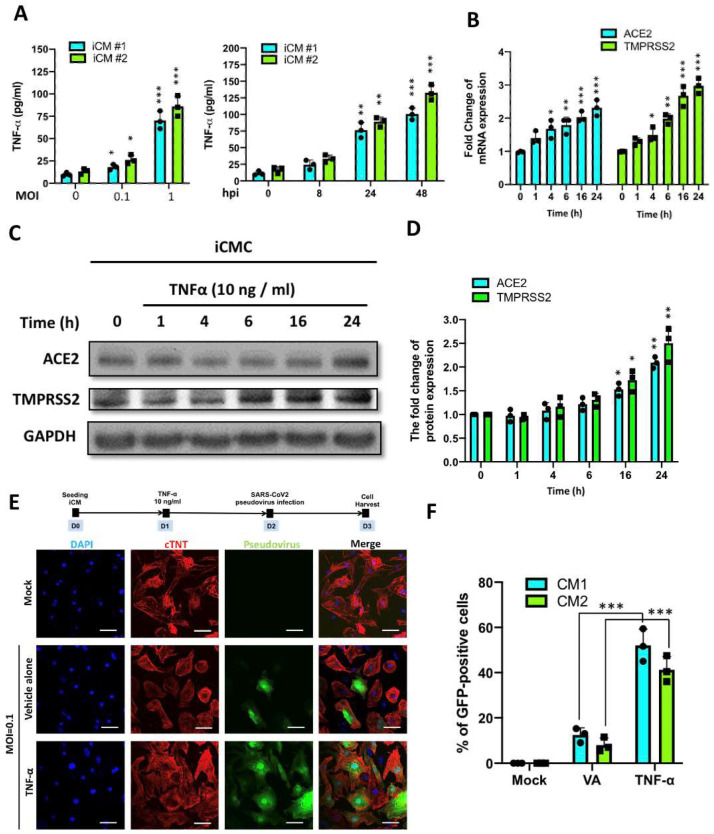
Effect of TNF-α on the entry of SARS-CoV2 pseudovirus in iCMs. (**A**) Effect of SARS-CoV2 infection on TNF-α secretion. iCMs were exposed to various MOI (left) of SARS-CoV2 or SARS-CoV2 (MOI = 1) for a different time, and then the secretion of TNF-α in the iCM culture was examined. (**B**) Quantitative real-time PCR showed that TNF-α (10 ng/mL) increased the gene expression of ACE2 and TMPRSS2 in iCMs in a time-related manner. * *p* < 0.05, ** *p* < 0.01, *** *p* < 0.001 vs. 0 h. (**C**) Western blot showed that TNF-α (10 ng/mL) increased the protein amount of ACE2 and TMPRSS2 in iCMs. (**D**) Quantifications of Western blots. (**E**) Upper panel: 24 h after seeding, iCMs were treated with TNF-α (10 ng/mL) for 24 h. After the TNF-α treatment, iCMs were infected with SARS-CoV2 pseudovirus. One day after the viral infection, iCMs were harvested and subjected to immunofluorescence staining. Lower panel: Immunofluorescence staining showed that the entry of SARS-CoV2 pseudovirus into iCMs was increased upon TNF-α treatment as compared with the treatment of the vehicle alone (VA). Scale bar = 50 μm. (**F**) Quantification of the immunofluorescence results. *** *p* < 0.001 vs. vehicle alone.

Based upon our bioinformatics analysis, several inflammation-related genes were upregulated following the viral infection, including the CXCL10, TNF-α, and interleukin 1α, etc. Since the elevated expression of TNF-α has been reported during the initial stages of SARS-CoV-2 infection and may serve as a key component of the cytokine storm in COVID-19 [21,25], we hypothesized that TNF-α may contribute to the aggravation of the COVID-19 manifestations. Based upon our data of TNF-α as an upregulating factor in SARS-CoV2-infected iCMs, we next examined the consequence of SARS-CoV2 infection on TNF-α secretion. We exposed iCMs to various MOI of SARS-CoV2 or SARS-CoV2 (MOI = 1) for a different time and then examined the secretion of TNF-α in the culture of iCMs (Figure 5A). Our data revealed that SARS-CoV2 promoted TNF-α secretion in both MOI- and time-dependent manners. Next, we examined the effect of TNF-α treatment on the expression of ACE2 and TMPRSS2 in iCMs using qRT-PCR (Figure 5B). The exposure of iCMs to TNF-α (10 ng/mL) for different time periods resulted in a time-dependent increase in ACE2 and TMPRSS2 expression (Figure 5B). In addition, the Western blot data further confirmed the upregulation of ACE2 and TMPRSS2 proteins after the TNF-α treatment (Figure 5C,D). SARS-CoV2 pseudovirus is an artificial virus that carries the S protein of SARS-CoV2 and mimics the SARS-CoV2 entry process in target cells [28]. To examine whether the increase of ACE2 and TMPRSS2 protein contribute to the exacerbation of inflammatory response, we then investigated the viral entry of SARS-CoV2 pseudovirus in iCMs. The SARS-CoV2 pseudovirus was generated by transfecting the 293T cells with a lentiviral backbone plasmid encoding a fluorescent reporter protein and the spike (S) protein. Therefore, the entry of SARS-CoV-2 pseudovirus into the host cells could be tracked by detecting GFP signal. One day after the seeding, iCMs were treated with TNF-α (10 ng/mL) for the additional period of 24 h. After the TNF-α treatment, these iCMs were subjected to SARS-CoV2 pseudoviral infection for 24 h, followed by harvesting of iCMs and immunofluorescence staining (Figure 5E, upper). As demonstrated by the incorporation of the fluorescent signal, TNF-α treatment significantly increased the entry of SARS-CoV-2 pseudovirus into the host cells, as compared with iCMs treated with vehicle alone (Figure 5E,F). Collectively, these data indicated that TNF-α upregulates SARS-CoV2 entry receptors and exacerbates SARS-CoV2 pseudoviral infection in iCMs.

**Figure 6:**
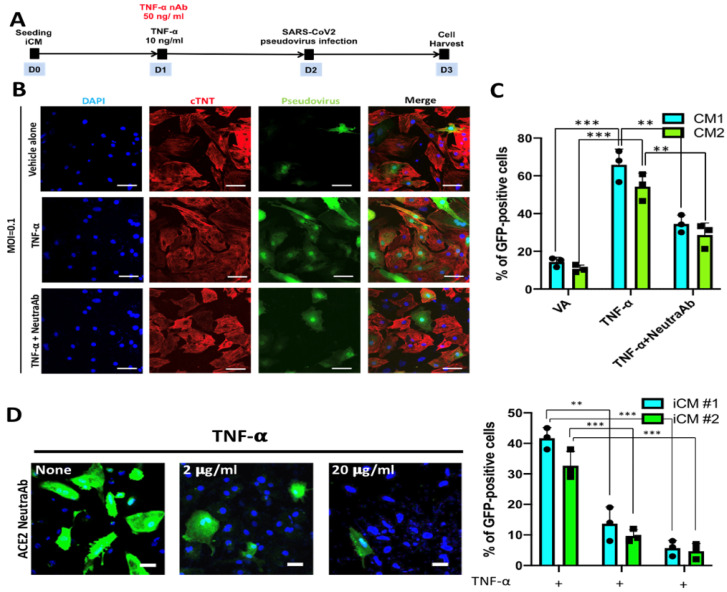
Neutralization of TNF-α suppressed the enhanced SARS-CoV2 pseudoviral entry in iCMs. (**A**) 24 hours after iCM seeding, the cells were co-treated with TNF-α (10 ng/mL) and TNF-α neutralizing antibody (TNF-α nAb; 50 ng/mL) for another 24 h. Then, iCMs were infected with SARS-CoV2 pseudovirus. At the end of the experiment course, iCMs were harvested and subjected to immunofluorescence staining. (**B**) Immunofluorescence staining showed the entry of SARS-CoV2 pseudovirus into iCMs was increased upon TNF-α treatment, as compared with vehicle alone (VA). The co-incubation of TNF-α and TNF-α nAb abrogated the enhanced viral entry. Scale bar = 50 μm. (**C**) Quantification of the immunofluorescence results. (**D**) Immunofluorescence staining showed the TNF-α-stimulated entry of SARS-CoV2 pseudovirus can be abrogated by ACE2 neutralization (Left). TNF-α-treated iCMs were preincubated with ACE2 neutralizing antibody at different doses (2 μg/mL or 20 μg/mL) for 16 h, and these cells were then subjected to SARS-CoV2 pseudoviral infection and further analysis. The quantifications of the immunofluorescence results. ** *p* < 0.01, *** *p* < 0.001 vs. vehicle alone.

To further validate the effect of TNF-α on the viral entry of SARS-CoV2 pseudovirus in iCMs, the TNF-α neutralizing antibodies (TNF-α nAb; 50 ng/mL) were simultaneously added into the culture to neutralize the effect of TNF-α (Figure 6A). We used confocal microscopy to evaluate SARS-CoV2 pseudovirus entry in iCMs by examining the fluorescence reporter protein of SARS-CoV2 pseudovirus. iCMs were harvested and subjected to immunofluorescence staining followed by co-incubating with TNF-α, TNF-α nAb, and the SARS-CoV2 (Figure 6A). The results showed that TNF-α increased the entry of SARS-CoV2 pseudovirus, and this phenomenon can be significantly reversed by the co-treatment of TNF-α nAb (Figure 6B,C). Furthermore, preincubation of iCMs with ACE2 neutralizing antibody at different doses (2 μg/mL or 20 μg/mL) for a period of 16 h showed a dose-related effect to abrogate the increased SARS-CoV2 pseudoviral entry promoted by TNF-alpha (10 ng/mL). Along with the observations of TNF-alpha-mediated stimulatory effect on ACE2 expression, our data indicated that the TNF-alpha-mediated stimulatory effect on SARS-CoV2 pseudoviral entry is predominantly through the SARS-CoV2 receptor ACE2. Collectively, based upon the findings of TNF-α upregulation in SARS-CoV2-infected iCMs (Figure 4), TNF-α may serve as a critical factor to potentiate SARS-CoV2 infection and hence worsen the manifestations of COVID-19.

## 3. Discussion

COVID-19 is mainly known to cause viral pneumonia and a variety of extrapulmonary complications. Among the reported COVID-19 cases, a large number of patients showed underlying cardiovascular comorbidities and/or cardiac risk factors [29]. A high incidence of acute cardiac injury was reported in hospitalized COVID-19 patients in Wuhan, China [30]. Besides, the studies that enrolled a large number of hospitalized COVID-19 patients in Italy [31] and New York City [32] also showed the prevalence of myocardial injury and high in-hospital mortality [31,32]. These clinical findings implied a correlation between SARS-CoV2 infection and its cardiac involvement. Furthermore, SARS-CoV2 transcriptional activity was detected in the heart sample from COVID-19 patients, suggesting the heart as a target organ for the SARS-CoV2 infection [33]. According to previous studies on COVID-19 pathogenesis, The COVID-19-induced manifestations and complications have been mainly attributed to the hyperactivation of COVID-19-induced host immune response that often elicits inflammatory responses. This phenomenon may cause the deterioration of the cardiac functions in patients with cardiac comorbidities [34]. Mounting evidence also showed the association of COVID-19 infection with severe inflammatory response causing myocarditis, cardiac arrhythmias, and vascular inflammation [29]. Another meta-analysis study of COVID-19 patients further showed that pre-existing cardiovascular diseases were significantly correlated with poor prognosis and the use of invasive mechanical ventilation [35]. Interestingly, several cases supported the crucial role of cytokine storm and increased TNF-α levels in the SARS-CoV2 pathogenesis [21,22,23,24]. Patients with cardiomyopathy, heart failure, or other atherosclerotic lesions also exhibited elevated TNF-α levels [36,37,38]. Nevertheless, the impact of increased TNF-α levels on SARS-CoV2 infection per se remains unclear. In the present study, our data demonstrated that SARS-CoV2 infection promoted TNF-α expression and secretion. This elevated TNF-α levels, and subsequently increased the expression of ACE2 and TMPRSS2, leading to the enhanced SARS-CoV2 entry in cardiomyocytes.

**Figure 7:**
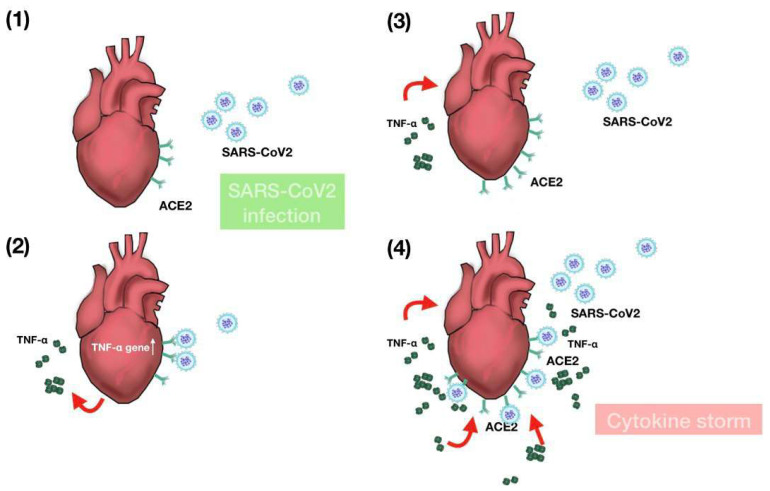
The enhanced SARS-CoV2 entry and TNF-α secretion may induce a cytokine storm in COVID19 patients. SARS-CoV2 binds to its ACE2 receptors in the heart (**1**) and increases the expression and secretion of TNF-α (**2**). The increased level of TNF-α upregulates ACE2 and TMPRSS2 expressions, resulting in the enhanced entry of SARS-CoV2 (**3**). The enhanced viral entry and TNF-α secretion may contribute to the cytokine storm (**4**).

Human iPSC-derived cardiomyocytes mimicking the electromechanical features of the heart have been utilized as the in vitro platform for investigating the SARS-CoV2 pathogenesis in the heart [18,39,40,41]. Consistent with the previous findings, [18] we found that iCMs express ACE2 and TMPRSS2, demonstrating the susceptibility of these cells to the SARS-CoV2 infection. Based on our RNA-seq data, TNF-α expression was elevated after SARS-CoV2 infection, consistent with the observations by other groups [41,42]. In addition to the genome-wide mRNA profiling, we also used X2K software to predict the kinases and transcription factors upstream of the identified inflammation-associated genes. The inferred networks of SARS-CoV2-upregulated inflammation-associated genes in iCMs were predicted to be constituted of kinases (HIPK2, ERK2, ERK1, CSNK2A1, MAPK1, and MAPK14) and transcription factors (NFIC, IRF1, CEBPD, IRF8, CREB1, RUNX1, RELA, STAT3, GATA2, and GATA1). A more in-depth analysis is required to investigate the key signaling pathways induced by SARS-CoV2 infection, and we further hypothesize that these findings may have implication for the development of adjuvant therapies for COVID-19 patients with cardiac inflammation. Furthermore, SARS-CoV2 infection downregulated the genes involved in NADH metabolic process, ATP metabolic process, and muscle contraction. NADH and ATP metabolic pathways are crucial for maintaining the regular cardiac metabolism and work output. Our data support the findings by Marchiano et al., demonstrating the cardiomyocyte metabolic disturbances in SARS-CoV2 infection followed by impaired electromechanical physiology [19]. In a nutshell, our findings showed that TNF-α enhanced viral entry of SARS-CoV-2, which in turn upregulated TNF- α, leading to a vicious circle of viral infection and the accumulation of the cytokines (Figure 7). The induction of TNF-α by SARS-CoV-2 infection and the subsequent upregulation of ACE2 and TMPRSS2 in cardiomyocytes may partially explain the high incidence of cardiac involvement in hospitalized COVID-19 patients [29,31,32]. Additionally, the recruitment of inflammatory cells by TNF-α and the increased susceptibility to further SARS-CoV-2 infection could exacerbate the viral infection and impair the metabolism, electrophysiology, and mechanical properties of the heart. Our data also suggested that the neutralization of TNF-α may be a therapeutic option to interrupt the vicious circle that links the viral infection to cytokine storm in COVID-19 patients with cardiac impairment.

## 4. Materials and Methods

### 4.1. Maintenance and Differentiation of Human-Induced Pluripotent Stem Cells

NTA is a subclone of the human-induced pluripotent stem cell (iPSC) line that was reprogrammed from peripheral blood, were collected following Ethical and Institutional Review Board of Taipei Veterans General Hospital (ID No. 2020-05-004C), using the integration-free Sendai virus carrying the four Yamanaka factors, as described in [43], and their genome integrity and pluripotency were evaluated. NTA was maintained in StemFlexTM Medium (Thermo Scientific, Waltham, MA, USA). NTA was sustained on precoated Geltrex^®^ Matrix (Thermo Scientific, Waltham, MA, USA) culture plates. hiPSCs were passaged every 5 to 6 days at 70% confluency using the nonenzymatic cell dissociation reagent Versene (Thermo Scientific, Waltham, MA, USA)-based protocol.

### 4.2. Differentiation of Cardiovascular Cells from hiPSCs

The protocol of differentiation of hiPSCs to cardiovascular cells was adapted with modifications from Halloin et al. [44]. The 70% confluent hiPSCs were detached and dissociated into small clumps with the Versene dissociation protocol and seeded in dishes. After kick-starting cardiac differentiation by 24 h of CHIR supplementation, WNT pathway attenuation was typically initiated after a 48- to 72-h gap. Pathway stimulator IWP2 was added on day 1 after 24 h CHIR treatment.

### 4.3. SARS-CoV-2 Virus Infection

SARS-CoV-2 coronavirus (isolate CDC04) was obtained from Taiwan Centers for Disease Control. All work involving live SARS-CoV-2 was performed in the Taiwan CDC-approved BSL-3 and BSL-4 facilities of the Institute of Preventive Medicine (IPM) in accordance with institutional biosafety requirements. Stocks of the virus were grown in Vero-E6 cells in DMEM supplemented with 2% fetal bovine serum (FBS; Thermo Fisher Scientific, Waltham, MA, USA). Virus-containing media was stored at −80 °C in single-use aliquots. Viral titers were performed using plaque assay as described [45]. The iPSC-derived cardiomyocyte cells were performed in the respective cell growth media and infected with SARS-CoV-2 for 24 h at 37 °C. At 24 hpi, cells were washed with PBS. For RNA analysis, the cells were lysed in TRIzol reagent. For immunofluorescence staining, the cells were fixed in 4% formaldehyde. For Western blot analysis, the cells were re-suspended in RIPA lysis buffer. The inactivated specimens were then removed from the BSL-3 for further analysis.

### 4.4. Infection with SARS-CoV-2 Pseudovirus

The SARS-CoV-2 pseudovirus construct was a gift from Dr. Yu-Chi Chou (National RNAi Core from Academia Sinica, Taiwan). The SARS-CoV-2 pseudovirus used a lentiviral as the backbone, a plasmid-encoding GFP sequence, a plasmid-expressing spike (S) protein as the surface envelope glycoprotein, and the minimal plasmid set of the lentiviral protein necessary to assemble viral particles (Tat, Gal-Pol, and Rev). The CMV promoter was used to drive the expression of GFP. To assay the pseudovirus infection, monolayer cultures were seeded in 6-well pseudovirus plates and were added at the indicated MOIs and centrifuged in the plates at 1200× *g* for 30 min. At 12 h post-infection, cells were washed three times with PBS; then, the infection medium was replaced with fresh medium and kept for 3 to 6 days. At 3 dpi and 6 dpi, cells were harvested for the qPCR assay or immunofluorescence analysis. For RNA analysis, cells were lysed in TRIzol.

### 4.5. Western Blot

Cells were lysed in RIPA buffer supplemented with protease inhibitor added (Merck Millipore, Billerica, MA, USA). The protein lysates were quantified and subjected to SDS-PAGE, followed by electroblotting onto PVDF membrane. The membranes were incubated with N protein of SARS-CoV-2 (GeneTex, GTX635689; GeneTex, CA, USA) or glyceraldehyde 3-phosphate dehydrogenase (GAPDH) antibodies (Cell Signaling Technology, Danvers, MA, USA), or ACE-2 antibodies (GenTex, GTX01160; GeneTex, CA, USA), or TMPRSS2 antibodies (abcam, ab109131; abcam, Cambridge, UK). The reactive bands were visualized by chemiluminescence detection reagents (Merck Millipore, Billerica, MA, USA).

### 4.6. Immunofluorescence

The monolayer cultures were washed twice in PBS and fixed in 4% paraformaldehyde for 15 min and then were permeabilized in 0.1% Triton X-100. After that, they were blocked with 5% fetal bovine serum (FBS; Thermo Fisher Scientific, Waltham, MA, USA) for 1 h at room temperature. Cells were incubated at room temperature for 2 h with primary antibodies (1:100 for anti-recoverin: Sigma-Aldrich AB5585, 1:100 for anti-CRX: Abcam AB140603, 1:100 for anti-HuC/HuD; abcam, Cambridge, UK: Thermo Scientific A-21271, 1:100 for anti-rhodopsin; Thermo Scientific, MA, USA: Abcam AB221664, 1:100 for anti-ACE-2; abcam, Cambridge, UK: GeneTex GTX01160, and 1:100 for anti-TMPRSS2; GeneTex, CA, USA: Abcam AB109131; abcam, Cambridge, UK). After 3 washes in PBS, the cells were then incubated with goat anti-rabbit secondary antibody (Alexa Fluor 594-conjugated) or donkey anti-mouse secondary antibody (Alexa Fluor 488-conjugated). The nuclei were counterstained with DAPI (blue) (Abcam). Cells were mounted on slides with a mounting solution.

### 4.7. Quantitative PCR and RT-PCR

The total RNA of samples was isolated by TRIzol reagent (Thermo Scientific, Waltham, MA, USA), according to the manufacturer’s protocols. RNA was quantified with NanoDrop 2000 (Thermo Scientific, Waltham, MA, USA); 2 micrograms of RNA were prepared for the reverse transcription reaction using SuperScript reverse transcription III (Invitrogen) to synthesize complementary DNA strands (cDNA). cDNA was used in the following quantitative PCR (qPCR) and RT-PCR. According to the product’s instructions, the qPCR reaction was performed using the Fast SYBR Green Master Mix (catalog number needed). qPCR analysis was performed using 3 independent biological and technical replicates. The primers were designed using the National Center for Biotechnology Information (NCBI) primer design tool, tested using a standard curve (information of primer sequences needed). Glyceraldehyde-3-phosphate dehydrogenase (GAPDH) was used as the housekeeping gene, and the relative gene expression of the target genes was determined using the 2^−∆∆^Ct (delta-delta Ct) method.

### 4.8. RNA-Seq

Total RNA was extracted using TRIzol reagent (Invitrogen, CA, USA) and resuspension in RNase-free water. The RNA-seq libraries of polyadenylated RNA were prepared using the TruSeq RNA Library Prep Kit V2 (Illumina, CA, USA), according to the manufacturer’s instructions. RNA-seq libraries for total ribosomal RNA-depleted RNA were prepared using the TruSeq Stranded Total RNA Library Prep Gold (Illumina, CA, USA), according to the manufacturer’s instructions. cDNA libraries were sequenced using an Illumina NextSeq 500 platform.

### 4.9. Bioinformatic Analyses

To cope with the wide range of transcripts, raw reads were aligned to the human genome using the RNA-Seq Alignment Application on Basespace (Illumina, CA, USA), following differential expression analysis using DESeq. Differentially expressed genes (DEGs) were characterized for each sample (*p*-adjusted value <0.05) and were used as a query to search for enriched biological processes and cellular components (gene ontology) and the network analysis of protein interactions using STRING. Heatmaps of the gene expression levels were constructed using the heatmap.2 function from the plot R package. Sparse principal component analysis (sPCA) was performed on the Log2 (fold change) values using SPC from the PMA package in R. Volcano plots were constructed using a custom script in R.

### 4.10. Statistical Analysis

Gene expression levels were calculated with relative expression levels (∆Ct). Data were expressed as mean ± standard deviation. Statistically significant differences between 2 groups or among multiple groups were detected by a paired Student’s two-tailed *t*-test and one-way ANOVA with Tukey’s post hoc, respectively, using SPSS Edition 25 software (Chicago, IL, USA). The criterion for significance was set as *p* < 0.05, and highly significant differences in the statistics were accepted if *p* < 0.001. All data presented are representative of at least 3 independents.

## Data Availability

The data presented in this study are available on request from the corresponding author.
